# Mouth Rinsing With a Pink Non-caloric, Artificially-Sweetened Solution Improves Self-Paced Running Performance and Feelings of Pleasure in Habitually Active Individuals

**DOI:** 10.3389/fnut.2021.678105

**Published:** 2021-05-12

**Authors:** Daniel R. Brown, Francesca Cappozzo, Dakota De Roeck, Mohammed Gulrez Zariwala, Sanjoy K. Deb

**Affiliations:** ^1^Department of Higher Education Sport, Loughborough College, Loughborough, United Kingdom; ^2^Centre for Nutraceuticals, School of Life Sciences, University of Westminster, Westminster, United Kingdom

**Keywords:** RPE, mouth rinse, placebo & nocebo effects, self-pace running, endurance, ergogenic aid

## Abstract

**Purpose:** The purpose of this study was to investigate whether mouth rinsing with a pink non-caloric, artificially sweetened solution can improve self-selected running speed and distance covered during a 30 min running protocol.

**Methods:** Ten healthy and habitually active individuals (six males, four females) completed two experimental trials in a randomised, single-blind, crossover design. Each experimental trial consisted of a 30 min treadmill run at a self-selected speed equivalent to 15 (hard/heavy) on the rating of perceived exertion scale. During exercise, participants mouth rinsed with either a pink or a clear non-caloric, artificially sweetened solution, with performance, perceptual and physiological measures obtained throughout.

**Results:** Self-selected running speed (+0.4 ± 0.5 km·h^−1^, *p* = 0.024, *g* = 0.25) and distance covered (+213 ± 247 m, *p* = 0.023, *g* = 0.25) during the 30 min running protocol were both improved by 4.4 ± 5.1% when participants mouth rinsed with the pink solution when compared to the clear solution. Feelings of pleasure were also enhanced during the 30 min treadmill run when participants mouth rinsed with the pink solution, with ratings increased from 3.4 ± 0.7 in the clear condition to 3.8 ± 0.6 in the pink condition (+0.4 ± 0.5, *p* = 0.046, *g* = 0.54).

**Conclusion:** Mouth rinsing with a pink non-caloric, artificially sweetened solution improved self-selected running speed, total distance covered, and feelings of pleasure obtained during a 30 min running protocol when compared to an isocaloric and taste-matched clear solution.

## Introduction

The effects of carbohydrate mouth rinse on running and cycling performance are well-documented in temperate conditions, with a 2–3% ergogenic benefit commonly reported during exercise bouts of ≤ 1 h duration (1–4). This ergogenic benefit is seemingly underpinned by the detection of a carbohydrate stimulus in the buccal cavity and the corresponding expectation of carbohydrate intake, which is thought to inhibit the negative afferent signals implicated in the aetiology of central fatigue (3). The reward and motor function areas of the brain are also activated, leading to improvements in pleasure, arousal and efferent motor output during high-intensity and/or prolonged exercise (4).

Additionally, the application of caffeinated mouth rinse has also received widespread attention in the literature (5–7). Although current data remains equivocal, with the heterogeneity of exercise protocols and caffeine doses suggested as potential factors (7), caffeinated mouth rinse is reported to improve cycling and arm cranking performance by 9–16% during exercise bouts of 30 min duration (5, 6). When compared to carbohydrate mouth rinse, it is the bitterness of caffeine that is detected in the buccal cavity which is suggested to underpin its ergogenic potential via an upregulation of sympathetic neural activity and corresponding physiological and efferent motor outputs during exercise (7).

The consumption of food and drink, however, is a multisensory experience, with colour often a characteristic that is manipulated to enhance subsequent taste perception and psychophysiological response (8). The perception of sweetness, for example, is known to be enhanced if a food or drink product is presented with a pink appearance, an outcome which is consistent across European, Asian and American cultures (9, 10). Although there may be no immediate link between drink colour, taste perception and performance nutrition, if the colour pink is associated with perceived sweetness (9, 10), and therefore expectations of sugar/carbohydrate intake (11), it may be plausible that the provision of a pink-coloured mouth rinse during exercise may elicit a similar ergogenic benefit to that of carbohydrate mouth rinse through a potential placebo effect.

The placebo effect can be described as the psychophysiological response to a purported beneficial treatment (12), with greater expectations of a positive outcome often leading to a greater placebo effect (13). In a recent review, a small to moderate effect size was reported for the placebo effect of nutritional ergogenic aids on subsequent exercise performance (*n* = 1,099, effect size = 0.35) (12). To date, however, the placebo effect of carbohydrate remains equivocal, with no ergogenic benefit reported in trained cyclists (*n* = 10) undertaking a ~ 1 h cycling time trial (following an initial 2 h preload at 50% W_max_) while consuming either a carbohydrate placebo solution (65.94 ± 5.56 min) or plain water (66.35 ± 6.15 min) (14). Conversely, a placebo effect was reported during a 40 km time trial in trained cyclists as being told that a non-caloric drink contained carbohydrate improved performance (+4.3 ± 4.8%, *n* = 8) when compared to being told the drink was a placebo (+0.5 ± 5.8%, *n* = 7) or not being told any information at all (−1.1 ± 8.5%, *n* = 7) (15). The disparity in performance outcomes was attributed to the metabolic importance of carbohydrate availability during exercise bouts of ≥ 1 h duration (4, 14). What remains unexplored, however, is whether a placebo effect of carbohydrate exists when the drink/solution is rinsed, rather than consumed, during exercise bouts of ≤ 1 h duration that are less likely to be constrained by carbohydrate availability.

As such, the current study implements a 30 min exercise protocol, adapted from previous literature conducted on carbohydrate mouth rinse (1), to investigate whether mouth rinsing with a pink non-caloric, artificially sweetened solution can improve self-selected running speed, distance covered and feelings of pleasure and arousal during submaximal exercise of ≤ 1 h duration. To help with pacing, participants were instructed to self-select the speed necessary to maintain a rating of perceived exertion (RPE) of 15 (hard/heavy) throughout. It was hypothesised that mouth rinsing with the pink solution would improve self-selected running speed and distance covered, an ergogenic benefit underpinned by enhanced feelings of pleasure and/or arousal during exercise.

## Methods

The methods for the current study are reported in accordance to the Proper Reporting of Evidence in Sport and Exercise Nutrition Trials (PRESENT) 2020 checklist (16).

### Design

A randomised, participant single-blinded, crossover experimental design was employed to test the effect of mouth rinse solution colour on exercise performance during a 30 min run anchored at an RPE of 15. All participants visited the laboratory (temperature: 21.9 ± 1.8°C; pressure: 754.6 ± 8.6 mmHg, humidity: 41.1 ± 13.7%) on three occasions separated by 1 week (one preliminary trial and two experimental trials) at a similar time of day (± 1.0 h). Randomisation of experimental trials was performed using an online randomising tool (Randomizer.org), which randomly allocated the order of experimental trials for each participant. This study received ethical approval from the University of Westminster local ethics committee (ETH1819-0745).

### Participants

Ten healthy and habitually active individuals (six males, four females; age: 30 ± 3 years, height: 175 ± 14 cm, body mass: 75 ± 17 kg; exercise frequency: ≥ 3 times·week^−1^) volunteered to participate in the study. Participants were eligible to volunteer in the study if they were over 18 years of age, regularly undertook weekly running exercise as part of their training regime and were deemed fit to exercise by the completion of a pre-activity readiness questionnaire. Individuals who were on medication for health-related conditions or were taking nutritional ergogenic aids, such as buffering agents, nitrates or creatine, were excluded from the study. Participants refrained from strenuous exercise and the consumption of alcohol and caffeine in the 24 h preceding each visit (17, 18). Habitual dietary intake was maintained; however, participants entered the laboratory in a 4 h postprandial state, except for the ingestion of water to ensure euhydration. Compliance with the above procedures was checked via 24 h dietary recall, with dietary intake replicated prior to each trial.

### Preliminary Trial

During the preliminary trial participants watched a video presentation that detailed the ergogenic benefits of carbohydrate mouth rinse, before being informed that the study aimed to compare the effects of mouth rinsing two commercial sports drinks. The use of the video presentation was to ensure that each participant received a standardised explanation of the study, but to also ensure that participants were blinded to the true aims of the study whilst participating. The participants were blinded from the content of the drinks (i.e., no nutritive or carbohydrate content) but were able to see the colour of the solutions in the experimental trial. Due to the nature of the study design it was not possible to blind the researcher, as they were aware of the true aims of the investigation.

Following the instructional video, the participants entered the laboratory for a full familiarisation of the protocol to ensure they were accustomed to procedures employed during each experimental trial. The familiarisation trial replicated the procedures of the experimental trial described below; however, the participants were asked to rinse their mouth with unsweetened clear water to familiarise them with the mouth rinsing protocol during the running trial. No feedback or information on performance was given to the participant following completion of the exercise protocol.

### Experimental Trials

Each experimental trial required participants to exercise for 44 min on a motorised treadmill (h-p cosmos, Germany), which was set at a gradient of 1.0% to replicate outdoor running (19). Adapted from similar research conducted previously (1), participants undertook a 12 min warm-up consisting of a 2 min walk at 4 km·h^−1^ and a 10 min run at a self-selected speed equivalent to 12 (between light and somewhat hard) on the RPE scale (20). This self-selected speed was recorded and replicated during the second experimental trial. Following the warm-up, participants then completed the 30 min self-paced running protocol, with participants instructed to self-select the speed necessary to maintain an RPE of 15 (hard/heavy) throughout. Information regarding time remaining, time until the next mouth rinse and prompts to ensure RPE remained at 15 were received, with no other information or external encouragement provided. Distance covered (m) and mean speed (km·h^−1^) were recorded for both the entire 30 min and at 5 min intervals during the protocol. Heart rate, felt arousal (6 point scale; 1 = low arousal, 6 = high arousal) (21) and pleasure/displeasure (11 point bipolar scale; −5 = very bad, 0 = neutral, +5 = very good) (22) were measured 30 s prior to each mouth rinse (as detailed below), as well as on run completion. Following the completion of the second experimental trial, participants were interviewed to determine perceived differences in distance covered and drink ratings between trials. Participants were then informed about the true aims of the study, with the requirement for deception/blinding explained in full. An opportunity to ask any questions pertinent to the study was also provided.

### Intervention

During the two experimental trials, participants rinsed with one of two randomly assigned solutions. Each solution was prepared identically by adding 0.12 g of pure sucralose (Bulk Powders, United Kingdom) to 500 mL of plain water. One solution, however, had no colourant added (clear), whereas the other had two drops of non-caloric pink colourant added (Waitrose Limited, United Kingdom) to achieve the desired pink appearance (pink). Each drink was stored at ~4°C before being decanted into a clear plastic cup on the day of each experimental trial. Despite being identical apart from colour, a pilot study conducted prior to the current investigation reported that participants (*n* = 18) preferred the pink solution, perceiving it as both sweeter (+0.61, *p* = 0.002) and tastier (+0.56, *p* = 0.008) on a 5-point Likert scale (1 = strongly disagree, 5 = strongly agree).

Participants were provided with 25 mL of solution (pink or clear) and instructed to rinse it around the oral cavity for 5 s before expectorating it into a pre-weighed plastic container. Each plastic container was weighed pre- and post-expectoration to assess whether participants had ingested any of the solution. Mouth rinse solution (pink or clear) was provided on 9 occasions during each experimental trial; four times during the warm-up (immediately post-2 min walk at 4 km·h^−1^ and at min 3, 6, and 9 of the 10 min run at RPE 12) and on five occasions during the 30 min run (min 5, 10, 15, 20, and 25, respectively).

### Statistical Analysis

As assumptions of normality and homogeneity were met, a paired *t*-test was used to compare differences between conditions in total distance covered and mean speed throughout the entire 30 min exercise test. A *t*-test was also performed to detect whether a trial order effect was present. A two-way (condition × time) repeated measures analysis of variance (ANOVA) was used to determine differences in distance covered, mean speed, perceptual variables and mean heart rate during each 5 min interval of the 30 min run. *Post hoc* analysis with a Bonferroni adjustment was performed where significant interactions were observed to determine any significant differences between the 5 min segments. Effect sizes were calculated using Hedge's *g* and were interpreted as trivial (<0.20), small (0.20–0.49), moderate (0.50–0.79) or large (≥0.80) (23). Confidence intervals (CI) (±95.0%) were also calculated and are reported where necessary. Descriptive data are displayed as mean ± standard deviation (SD). To establish statistical power, a *post hoc* power calculation was performed. Statistical analysis was conducted using a statistical software package (SPSS, Version 26, USA), with significance accepted at *p* < 0.05.

## Results

**Figure 1:**
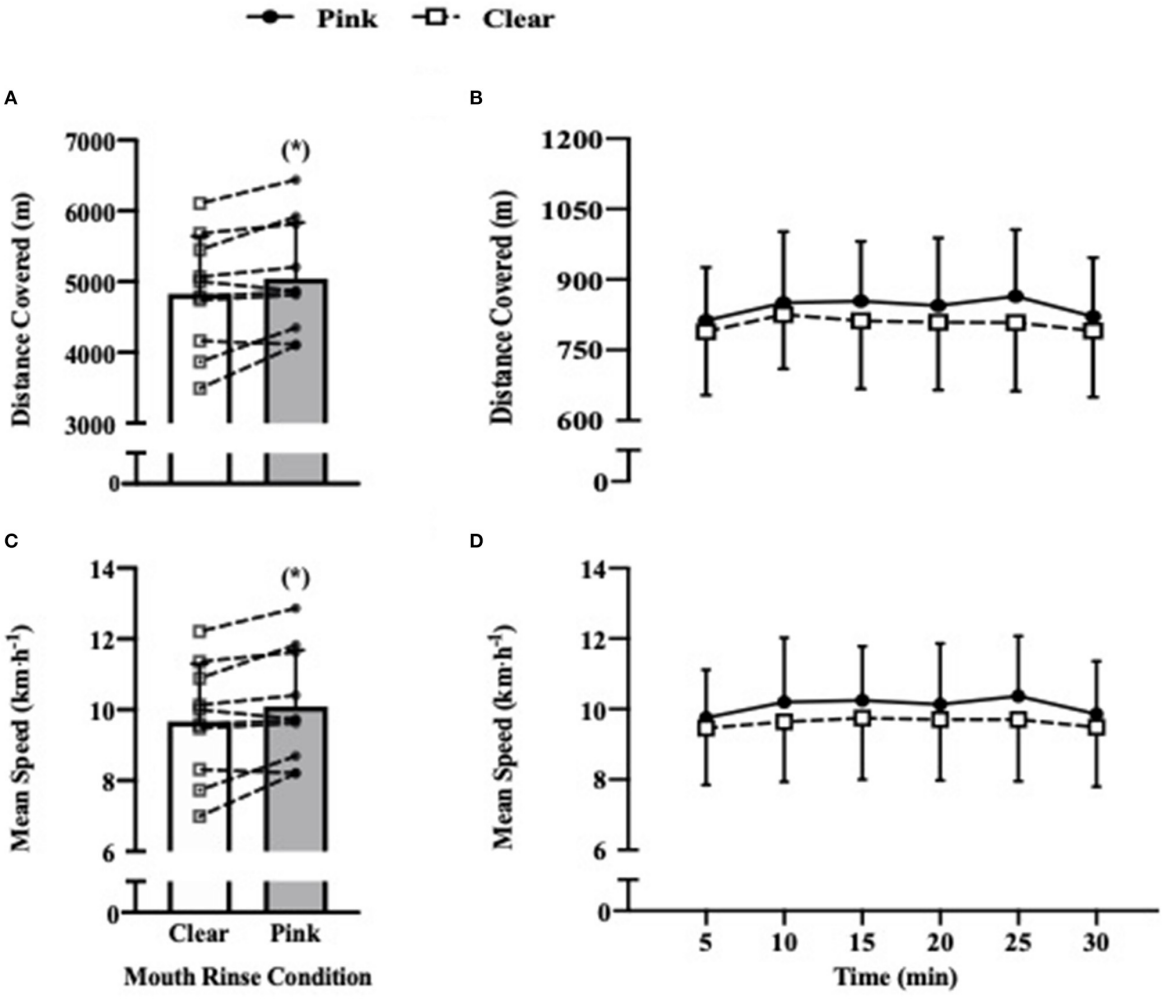
Shows bar charts that represent overall mean differences between conditions during the full 30 min protocol with lines illustrating individual responses in distance covered **(A)** and mean speed **(C)**. The five min splits of mean distance covered **(B)** and mean speed **(D)** are shown to depict general pacing profile between conditions. ^*^Denotes *p* < 0.05 between conditions.

Total distance covered during the 30 min self-paced running protocol (Figure 1A) was improved from 4,835 ± 816 m in the clear mouth rinse condition to 5,047 ± 795 m in the pink mouth rinse condition, which equates to a 4.4 ± 5.1% improvement (+213 ± 247 m, 95.0% CI = 36–389 m, *p* = 0.023, *g* = 0.25). Mean speed (Figure 1C) was also improved from 9.7 ± 1.6 km·h^−1^ in the clear mouth rinse condition to 10.1 ± 1.6 km·h^−1^ in the pink mouth rinse condition, which again equates to a 4.4 ± 5.1% improvement (+0.4 ± 0.5 km·h^−1^, 95.0% CI = 0.1–0.8 km·h^−1^, *p* = 0.024, *g* = 0.25). No trial order was present when comparing total distance covered (*p* = 0.570). There was also no (condition × time) interaction observed across each 5 min interval for either distance covered (*p* = 0.413; Figure 1B) or mean speed (*p* = 0.412; Figure 1D), suggesting that the general pacing profile of the 30 min run was similar across both mouth rinse conditions. The *post hoc* power analysis reported a power (1 – β) of 0.68 for distance covered and 0.61 for mean speed.

**Figure 2:**
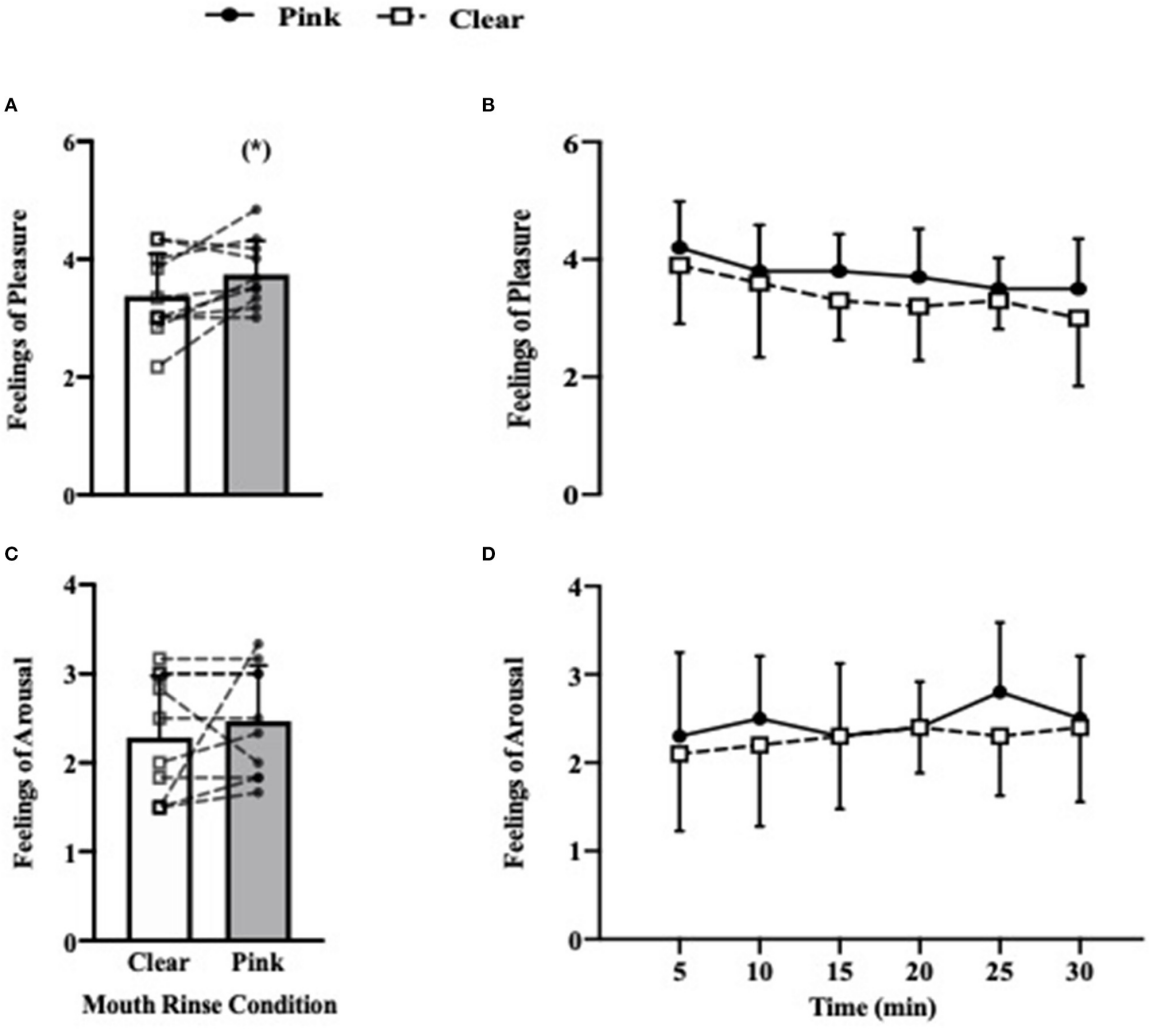
Shows bar charts that represent overall mean differences between conditions during the full 30 min protocol with lines illustrating individual responses in pooled feelings of pleasure **(A)** and pooled arousal **(C)**. **(B,D)** Show the five min splits of mean feelings of pleasure and mean arousal, respectively. ^*^Denotes *p* < 0.05 between conditions.

Feelings of pleasure were enhanced during the 30 min self-paced running protocol when participants mouth rinsed with the pink solution (Figure 2A), with ratings increased from 3.4 ± 0.7 in the clear mouth rinse condition to 3.8 ± 0.6 in the pink mouth rinse condition (+0.4 ± 0.5, 95.0% CI = 0.0–0.7, *p* = 0.046, *g* = 0.54). There was no effect of time (*p* = 0.061) or a (condition × time) interaction (*p* = 0.650) observed for this variable (Figure 2B). Feelings of arousal, however, were not influenced by mouth rinse colour, with no differences reported between mouth rinse conditions during the 30 min self-paced running protocol (pink: 2.5 ± 0.6 vs. clear: 2.3 ± 0.7, *p* = 0.405; Figure 2C). There was also no effect of time (*p* = 0.408) or a (condition × time) interaction (*p* = 0.058) observed for this variable (Figure 2D).

Heart rate increased progressively over time during the 30 min self-paced running protocol (+17 ± 9 beats·min^−1^; *p* < 0.001). There were, however, no differences observed between the pink and clear mouth rinse conditions (pink: 154 ± 15 beats·min^−1^ vs. 152 ± 19 beats·min^−1^, *p* = 0.386). There was also no (condition × time) interaction observed for this variable (*p* = 0.372). Post-trial interview responses report that all participants thought the pink solution was sweeter, while six of the 10 participants also thought they ran further within the pink mouth rinse condition when compared to the clear mouth rinse condition. Of these six participants, five actually ran further during the trial by 261 ± 250 m.

## Discussion

The current study is the first to report an ergogenic benefit of mouth rinse colour during submaximal exercise of ≤ 1 h duration. Indeed, mouth rinsing with a pink non-caloric, artificially sweetened solution improved self-selected running speed at an RPE of 15 and subsequent distance covered during a 30 min self-paced running protocol when compared to an isocaloric and taste-matched clear solution. This equates to a mean 0.5 km·h^−1^, 213 m or 4.4% improvement in performance. An increase in feelings of pleasure was also reported during exercise in the pink mouth rinse condition, a potential psychophysiological mechanism which may have underpinned the performance improvement reported.

The influence of colour on athletic performance has received interest previously, ranging from correlations between the colour of an athlete's or a sports team's uniform and sporting success (24, 25), to more causal relationships whereby the colour red has been reported to increase the feeling of anger (26), the concentration of testosterone (27) and even the generation of muscular power during exercise of differing intensities (28). Additionally, the role of colour in gastronomy has received widespread interest, with a vast array of cross-cultural research published on how visual cues or colour can affect subsequent flavour perception when eating and drinking (8–10). An interesting and novel finding from the current study seems to combine the art of gastronomy with performance nutrition, as adding a pink colourant to a non-nutritive, artificially sweetened solution not only enhanced the perception of sweetness (as quantified in the pilot study and confirmed in the post-trial interviews), but also enhanced feelings of pleasure, self-selected running speed and distance covered during a 30 min run. It is clear that future research is required to explore this potential relationship in further detail.

The performance findings of this study are also consistent with those reported in trained runners (*n* = 10) undertaking a similar 30 min self-paced running protocol, whereby mouth rinsing with a 6.0% carbohydrate solution improved self-selected running speed (+0.3 km·h^−1^) and total distance covered (+115 m) by 1.7% when compared to a placebo (1). Despite mouth rinse being matched for colour, viscosity and taste between conditions, this performance improvement was also seemingly underpinned by enhanced feelings of pleasure at the onset of exercise in the carbohydrate mouth rinse condition and the corresponding increase in self-selected running speed over the first 5 min of the 30 min running protocol (1). Although the general pacing profile in the current study was reported as similar across both mouth rinse conditions, the overall improvement in self-selected running speed (0.5 km·h^−1^) and total distance covered (+213 m) in the pink mouth rinse condition was paired with enhanced feelings of pleasure during exercise, highlighting further similarities to the outcomes reported by Rollo et al. (1).

The performance effect sizes reported in this study (small; *g* = 0.25 for both distance covered and mean speed, respectively) are also consistent with those reported in two recent reviews, conducted with the purpose of quantifying the effect of carbohydrate mouth rinse on time trial performances (29, 30). A small effect of carbohydrate mouth rinse (effect sizes = 0.25), for example, was reported for running and cycling time trials of ≥ 25 min duration (29), as well as for power generated during cycling time trials only (30). Interestingly, a 1.3% boost in performance was also suggested if time trials were performed following a 10 h fast rather than a 2 h fast (31), an outcome potentially explained by the more intense activation of the central reward systems of the brain when in a fasted state (32). In the current study, as exercise commenced in a 4 h fasted state, it remains unclear as to whether the small, yet significant, ergogenic benefit of mouth rinse colour would be augmented or mitigated under different dietary conditions. As athletes usually load with exogenous carbohydrates to maximise endogenous glycogen stores prior to competition (33, 34), the influence of prior dietary intake on the ergogenic potential of mouth rinse solution/drink colour clearly requires further investigation moving forwards.

Possible explanations that may underpin the “feel good” effect and corresponding ergogenic benefit reported in the pink mouth rinse condition are received from previous exploratory research. The detection of carbohydrates (i.e., glucose or maltodextrin) in the buccal cavity, for example, are reported to activate the central reward systems of the brain, such as the anterior cingulate cortex and ventral striatum, that are believed to mediate positive emotional, cognitive and behavioural responses to this stimuli (35, 36). In the current study, as a non-caloric artificial sweetener (sucralose) was added to both mouth rinse solutions, a carbohydrate stimulus was not present. As such, it may be plausible to suggest that the “feel good” effect and corresponding ergogenic benefit reported in the pink mouth rinse condition may have been underpinned by a placebo effect, with the colour pink often associated with sweetness and thus expectations of sugar/carbohydrate intake (11). This hypothesis is supported by pilot study data (*n* = 18), as the pink mouth rinse solution was perceived as both sweeter (+0.61) and tastier (+0.56) than the clear mouth rinse solution using a 5-point Likert scale (1 = strongly disagree, 5 = strongly agree). Future exploratory research is necessary to elucidate whether this enhanced perception of sweetness remains present in exercising participants and whether the proposed placebo effect causes a similar activation to the reward areas of the brain that are commonly reported following the actual provision of a carbohydrate stimulus.

As the current study implemented a self-paced exercise protocol adapted from previous literature conducted on carbohydrate mouth rinse (1), participants were asked to run at a speed equivalent to an RPE of 15 (hard/heavy) rather than complete a set distance in the fastest time possible. Although this provides an insight into how mouth rinse colour can influence self-selected running speed, it does not allow for the collection of data pertaining to perceptions of fatigue and effort that often underpin subsequent improvements in exercise performance (37). Nevertheless, with RPE and ratings of pleasure-displeasure having a strong and inverse relationship (38), the enhanced feelings of pleasure reported in the pink mouth rinse condition may have reduced the RPE for a given running speed and potentially explain the subsequent improvements in self-selected running speed and distance covered observed in the current study. Indeed, the influence of colour on perceptions of effort during exercise has been demonstrated previously, with RPE reported as lower if exercise (5 min cycle at 50% W_max_, *n* = 14) was performed while watching video footage of a rural cycling course (11.1 ± 1.6) when compared to if an achromatic filter (12.1 ± 1.6) or a red filter (12.3 ± 1.6) was applied (26). Future research should, therefore, seek to investigate whether the colour of a mouth rinse solution can provide a similar benefit to perceptions of fatigue and effort during the completion of ecologically valid performance time trial events.

As the intra-individual variation in performance was not quantified in the current investigation, the reproducibility of the 30 min treadmill protocol is inferred from previous literature in recreationally active males (*n* = 10) (39). In this study, a full familiarisation and three experimental 30 min treadmill time trials were conducted across a 2-week period, with an intra-individual variation of 1.5 and 1.6% reported between experimental trials one and two and experimental trials two and three, respectively. An intra-individual variation of 3.8% was also noted between the full familiarisation and experimental trial one, indicating the importance of a single familiarisation session prior to the first experimental trial (39). Although the current study was anchored at an RPE of 15 (hard/heavy), following a single familiarisation session performance was improved by 4.4% in the pink mouth rinse condition when compared to the placebo. To ensure that changes in performance can be confirmed as meaningful, future research should seek to calculate intra-individual variation within the actual sample recruited. Another potential limitation is the lack of menstrual cycle control in the female participants recruited to this study (*n* = 4). Despite this, it is only the early follicular phase of the menstrual cycle that is suggested to have a trivial effect on exercise performance, with no differences reported between all other phases (40). Nevertheless, future investigations may wish to monitor and control for this potential trivial impact when conducting exercise performance research within a female population. Finally, the *post hoc* power calculation suggested this study was underpowered; however, care should be taken when interpreting *post hoc* power calculations as they have been suggested to not reflect the true statistical power of a study (41). Future research should endeavour to conduct a *priori* sample size calculation to substantiate the outcomes of this investigation.

The current study highlights the potential influence mouth rinse solution/drink colour can have on both perceptual and performance measures obtained during subsequent exercise performances. This novel finding should emphasise the methodological importance of ensuring and reporting colour matching, with 3 of 11 (7), 8 of 25 (29) and 4 of 13 (30) studies analysed in recent mouth rinse reviews not explicitly providing this information. Although maltodextrin is often used as a colourless source of carbohydrate within the carbohydrate mouth rinse literature (29, 30), if an ergogenic benefit of mouth rinse solution/drink colour is possible, colour matching should be of utmost importance when designing and implementing future research methodologies within this subject area.

To conclude, mouth rinsing with a pink non-caloric, artificially sweetened solution improved self-selected running speed and total distance covered during a 30 min run at an RPE of 15 in habitually active participants. The improvement in self-selected running speed and total distance covered was also paired with an increase in feelings of pleasure reported by participants during exercise. Future research should seek to elucidate the link between mouth rinse colour, perceived carbohydrate intake and psychophysiological outcomes in exercising humans.

## Data Availability Statement

The raw data supporting the conclusions of this article will be made available by the authors, without undue reservation.

## Ethics Statement

The studies involving human participants were reviewed and approved by College of Liberal Arts and Sciences Ethics committee, University of Westminster. The patients/participants provided their written informed consent to participate in this study.

## Author Contributions

Material preparation, data collection, and analysis were performed by FC, DD, MZ, and SD. The first draft of the manuscript was written by DB, FC, and SD, with all authors contributing to draft versions. All authors contributed to the study conception and design, read, and approved the final manuscript.

## Conflict of Interest

The authors declare that the research was conducted in the absence of any commercial or financial relationships that could be construed as a potential conflict of interest.
